# Molecular classification of urothelial bladder carcinoma

**DOI:** 10.1007/s11033-023-08689-7

**Published:** 2023-07-31

**Authors:** Lucia Schwarzova, Zuzana Varchulova Novakova, Lubos Danisovic, Stanislav Ziaran

**Affiliations:** 1 Institute of Medical Biology, Genetics and Clinical Genetics, Faculty of Medicine, Comenius University, Bratislava, Slovakia; 2 Department of Urology, Faculty of Medicine, Comenius University, Bratislava, Slovakia

**Keywords:** Urothelial bladder carcinoma, Molecular subtypes of bladder tumors, NMIBC, MIBC, Expression

## Abstract

Urothelial bladder carcinoma (UC) ranks among the top ten most commonly diagnosed cancers worldwide on an annual basis. The standardized classification system for urothelial bladder tumors is the Tumor, Node, Metastasis classification, which reflects differences between non-muscle-invasive bladder carcinoma (NMIBC) and muscle-invasive bladder carcinoma (MIBC) and it depends on the extent to which tumor has infiltrated the bladder wall and other tissues and organs. NMIBC and MIBC exhibit great intrinsic heterogeneity regarding different prognoses, survival, progression, and treatment outcomes. In recent years, studies based on mRNA expression profiling revealed the existence of biologically relevant molecular subtypes of UC, which show variant molecular features that can provide more precise stratification of UC patients. Here, we present a complex classification of UC based on mRNA expression studies and molecular subtypes of NMIBC and MIBC in detail with regard to different mRNA expression profiles, mutational signatures, and infiltration by non-tumor cells. The possible impact of molecular subtyping on treatment decisions and patients’ outcomes is outlined, too.

## Introduction

Among men in Europe, urothelial bladder carcinoma (UC) ranked as the fourth most frequently diagnosed type of cancer in 2020. Of the 2.1 million new cancer cases in that year, UC represented 7.3% [1]. UC is the prevailing histological type of the disease, and for clinical and treatment purposes, it can be categorized into two types, non-muscle-invasive bladder carcinoma (NMIBC) and muscle-invasive bladder carcinoma (MIBC), which are differentiated based on the extent of tumor growth into the bladder wall. Among newly detected bladder tumors (BT) cases, around 70% are classified as NMIBC, and 30% are classified as MIBC [2]. Although patients with NMIBC have better survival and prognosis, they have a higher risk of recurrence. Approximately 20% of NMIBCs progress to the more-aggressive MIBC [3]. Accurate determination of the stage of the disease is crucial for clinical diagnosis and prognosis. For this purpose, the Tumor, Node, Metastasis (TNM) classification system was established (Table 1) [4].

**Table 1:** The TNM classification of BT

	NMIBC			MIBC				
Stage	Ta	T1	Tis	T2		T3	T4	
				T2a	T2b		T4a	T4b
Localization of BT in bladder wall and beyond	mucosa	Submucosa/lamina propria	Carcinoma in situ	Muscle layer -superficially	Muscle layer -deeply	Perivesical fat	Prostate, uterus,vagina, intestines	Abdominal/pelvic walls

Transurethral resection (TUR-BT) combined with intravesical therapy is the usual treatment for NMIBC patients [5]. Due to the high rate of recurrence, these patients must also undergo regular cystoscopy. The treatment of MIBC involves administering neoadjuvant chemotherapy before performing radical cystectomy surgery with lymph node dissection and urine diversion [6].

Molecular classification of UC by mRNA expression profiling has emerged in recent years. This approach has provided new insights into UC classification, as mRNA expression signatures can be used to define several distinct molecular subtypes. Several research groups have proposed molecular classification of UC. For example, some of these researchers analyzed only NMIBC [7, 8], whereas others examined only MIBC [9, 10], and others examined both types [11–13]. Subsequent studies attempted to create a consensus classification by combining previous nomenclature [14]. Stratification of patients into more-detailed groups than just MIBC or NMIBC is crucial. Patient outcomes, reactions to therapy, and prognosis differ based on the subtype of UC [9–11, 15–17]. Herein, we describe a detailed and up-to-date molecular classification of UC that has the potential to influence treatment and thus prognosis in the near future.

## Novel molecular classification of MIBC

The earliest studies on this topic described two basic subtypes of MIBC. A research group at Lund University was the first to identify two intrinsic molecular subtypes of MIBC [18]. Damrauer and colleagues used consensus clustering to identify two distinct molecular subtypes of MIBC, luminal and basal [19]. Basal and luminal UC exhibit differences in transcriptional activity and enrichment of transcriptional factors that mediate the expression of basal or luminal biomarkers and cell differentiation. Expression of transcriptional factors such as *ΔNp63α* [9, 20], *STAT3* [21, 22], and epidermal growth factor receptor (*EGFR*) [9, 15, 19] is typical for basal tumors, whereas luminal tumors exhibit expression of *ESR1*, *TRIM24*, *GATA3*, *FOXA1* [23, 24], uroplakins, peroxisome proliferator activator receptor–gamma (*PPARγ*) and there are typical *FGFR3* mutations and expression [9, 15, 19].

Pioneering studies that described two subtypes of MIBC were advanced using a different approach based on global mRNA expression profiles of UC, which allowed for the identification of more subtypes. Hence, UC was divided into additional groups beyond the basal and luminal subtypes, and within these two types, there is heterogeneity. Researchers at Lund University expanded their original study of two intrinsic subtypes of MIBC and developed a new classification system including NMIBC and MIBC. This newer study identified five major subtypes: urobasal A (uroA), genomically unstable (GU), urobasal B (uroB), squamous cell carcinoma–like (SCCL), and infiltrated subtype which was believed to represent a heterogenous class of other tumor subtypes infiltrated by non-tumor cells [11]. Research within the Lund group continued and presented an updated classification consisting of 5 subtypes: urothelial-like (which can be further divided into UroA, UroB, UroC), genomically unstable (GU), basal/squamous cell carcinoma-like (Ba/Sq), mesenchymal-like (mes-like), and small cell/neuroendocrine-like (Sc/Ne) [12, 13]. A MD Anderson Cancer Center group identified three molecular subtypes of MIBC, luminal, basal, and p53-like [9]. The so-called TCGA group identified five expression subtypes of MIBC: luminal-papillary, luminal-infiltrated, luminal, basal-squamous, and neuronal [10]. As different groups used different nomenclature, Kamoun et al. proposed a consensus classification of MIBC by comparing six different MIBC subtype classifications and analyzing 1750 MIBC transcriptomic profiles. Their consensus classification system included six subtypes of MIBC: luminal papillary (LumP), luminal non-specified (LumNS), luminal unstable (LumU), stroma-rich, basal/squamous (Ba/Sq), and neuroendocrine-like (Ne-like) [14].

The three luminal subtypes (LumP, LumU, LumNS) typically exhibit a luminal morphology and overexpress *PPARγ, GATA3*, and *FOXA1*, which are urothelial differentiation markers. In addition, they also overexpress uroplakins such as *UPK1A* or *UPK2*, and *KRT20*, which are normally expressed in high levels in terminally differentiated urothelial cells, as well as receptors with regulatory factors involved in estrogen signaling (*ESR2*) (Table 2) [9, 10, 14, 25]. Apart from these similar features, the luminal subtypes do not share the same molecular signatures. Tumors of the LumP subtype are characterized by high *FGFR3* transcriptional activity [14], and they exhibit a noninvasive Ta pathway signature [26]. LumNS tumors display elevated infiltration of stromal cells, especially fibroblasts, and infiltration by immune cells, especially B and T lymphocytes. Aside from LumNS, tumors of no other luminal subtype show signs of immune infiltration. LumU tumors displayed the most significant cell cycle activity compared to other luminal subtypes [14].

**Table 2:** MIBC subtypes and their typical molecular features

	LumP	LumNS	LumU	Stroma-rich	Ba/sq	Ne-like
Differentiation	Luminal	Luminal	Luminal	Luminal and basal	Basal and squamous	Neuroendocrine
Gene expression	*PPARγ* *GATA3* *FOXA1* *UPK1A* *UPK2* *KRT20* *ESR2*	*PPARγ* *GATA3* *FOXA1* *UPK1A* *UPK2* *KRT20* *ESR2*	*PPARγ* *GATA3* *FOXA1* *UPK1A* *UPK2* *KRT20* *ESR2*	Markers of smooth muscle, endothelium, fibroblasts, myofibroblasts	*KRT5* *KRT6A* *KRT14* *CD44* *TGM1* *DSC3* *PI3*	Synaptophysin *CGA* *NSE/CD56*
Genomic alternation	*FGFR3* *KDM6A* *CDKN2A*	*ELF3* *PPARγ*	*PPARγ* *E2F3* *SOX4* *ERCC2* *TP53*		*TP53* or *RB1*	*TP53* and *RB1*
Infiltration	No	Stromal cells—fibroblasts, immune cells—B and T lymphocytes	No	Stromal cells Immune—T- and B-cell	Immune cells—Cytotoxic lymphocytes Natural killer cells	No
Prognosis	the best	Poor	Intermediate	Intermediate	Poor	The poorest

Typical *FGFR3* alternations in the LumP subtype include amplifications, overexpression, or *FGFR3-TACC3* fusions [10, 14]. Apart from *FGFR3* mutations, LumP tumors show frequent *KDM6A* mutations. *CDKN2A* deletions are present at the highest level in LumP tumors, which is in accordance with typical deletions of chromosome 9. The best overall survival (OS) is reported among patients with the LumP subtype (Table 2) [14]. It can be assumed that LumP subtype tumors progress through the papillary pathway and originate from class I NMIBC tumors that progressed to *FGFR3*-mutated tumors and then to LumP tumors [19]. The LumP subtype corresponds to UroA (Lund University group), and luminal-papillary (TCGA group) [14]. LumP subtype tumors characterized by the TCGA group exhibit retention of sonic hedgehog signaling [10] and low expression signature scores of carcinomas in situ (CIS) [27]. These tumors are of low stage and exhibit high purity (i.e., no, or little infiltration by non-tumor cells) [10].

LumNS tumors show enrichment in mutations affecting regulatory factors, for example, *ELF3* (which functions as an initial regulator of urothelium differentiation) and *PPARγ* [14] (by which *ELF3* is activated) [28]. Amplifications or fusions of *PPARγ* are common in LumNS subtype tumors. Within this subtype, increased infiltration by stromal and immune cells is reported (Table 2) [14]. The LumNS subtype resembles the epithelial-infiltrated subtype identified by Sjödahl et al., which is a combination of characteristics from the GU and Uro subtypes [12, 25].

Frequent *PPARγ* mutations are also reported in the LumU subtype, along with over-expression of *E2F3* and *SOX4.* The mutational signature typical of the LumU subtype includes mutations affecting the core component of the nucleotide-excision repair pathway, *ERCC2,* and mutations in *TP53* (Table 2). LumU is considered the most genomically unstable and altered class, as it displays the highest load of APOBEC-induced mutations among all luminal subtypes [14]. LumU corresponds to luminal (MD Anderson group) and GU (Lund University group) tumors [14]. LumU tumors are thought to originate from class II NMIBC tumors that progress via a non-papillary pathway. Thus, LumP and LumU tumors differ biologically and exhibit a dissimilar origin, which explains why these two types of tumors cannot be assessed and treated as the same [25].

The Ba/Sq subtype of MIBC is characterized by overexpression of *KRT5, KRT6A*, *KRT14*, and *CD44*, each of which represents a basal or stem cell marker. Within this group, enrichment in squamous differentiation markers has also been reported, such as *TGM1, DSC3*, and *PI3* (Table 2) [10]. An association with *HIF1A* regulon activity [14] and a strong association with *STAT3* regulon activity [14, 29, 30] have been demonstrated in Ba/Sq subtype tumors. A connection with *EGFR* regulon activity has also been reported [14, 31]. Genes such as *GATA3, PPARγ*, and *FOXA1*, which are associated with differentiation of the urothelium, are downregulated in Ba/Sq subtype tumors. This subtype typically consists of higher-stage tumors, mostly stage T3 or T4 [25]. *TP53* or *RB1* represent two of the most commonly altered genes in the Ba/Sq subtype. The co-occurrence of these two mutations is not as frequent as the occurrence of mutations in just one of the genes [14]. Mutations in tumor suppressors are necessary for the invasion and progression of UC, but mutations in just one tumor suppressor are not sufficient [32]. Deletion of 3p14.2 also frequently occurs in the Ba/Sq subtype of MIBC. The Ba/Sq subtype corresponds to the basal-squamous (TCGA group), Ba/Sq (Lund University group), and basal (MD Anderson group) subtypes [14].

The stroma-rich subtype is characterized by stromal infiltration [33], intermediate levels of urothelial differentiation, and enrichment in gene signatures representing smooth muscle, endothelium, fibroblasts, and myofibroblasts (Table 2) [14]. The stroma-rich subtype corresponds to the luminal-infiltrated (TCGA group), and p53-like (MD Anderson group) subtypes [14]. The luminal-infiltrated type identified by the TCGA group shows overexpression of several immune markers (namely *PDCD1* [*PD-1*] or *CD274* [*PD-L1*]) [10]. Infiltration by immune cells is detected predominantly in two subtypes of MIBC, namely the Ba/Sq and stroma-rich subtypes. These subtypes are not infiltrated by the same immune cell types but rather different types of immune cells; cytotoxic lymphocytes and natural killer cells are present within Ba/Sq subtype, while stroma-rich tumors are enriched in T- and B-cell markers [14, 34]. Similar to the LumNS subtype, the stroma-rich subtype has not been as extensively characterized compared with the other subtypes. Progressing MIBC tumors consist of diverse cellular components, including immune and stromal cells, in addition to tumor cells, which make it difficult to conduct comprehensive gene expression analyses [25].

The neuroendocrine-like (Ne-like) subtype is the least common of all MIBC tumor groups. A typical sign of Ne-like subtype tumors is enrichment in markers of neuroendocrine differentiation, such as neuron-specific enolase (i.e., *CD56*), synaptophysin, and chromogranin A. In contrast to the Ba/Sq subtype, in which generally only the *TP53* or *RB1* gene is mutated rather than both, in the Ne-like subtype, altered forms of both of these genes co-occur (Table 2) [10, 12, 14]. There are reports of concurrent *TP53* and *RB1* inactivation [14], *E2F3* amplification, and *TP53* mutation [10] or co-occurrence of *TP53* and *RB1* deletions [14]. As is well known, the co-occurrence of two or more mutated tumor suppressor genes imparts a much higher risk of progression and results in a worse outcome compared with tumors in which just one tumor suppressor gene is mutated. High cell cycle activity is another typical sign of Ne-like tumors. This subtype results in the worst OS [10]. The Ne-like subtype corresponds to the neuronal subtype identified by the TCGA group and the small cell/neuroendocrine-like subtype identified by Lund group [14].

## Novel molecular classification of NMIBC

Hedegard et al. conducted an extensive study on the transcriptional profile of 460 NMIBC tumors, of which 345 were Ta stage, 112 were T1 stage, 3 were CIS, and an additional 16 were MIBC tumors. They divided these tumors into 3 subclasses, named simply class I, II, and III. Tumors of high grade and stage and tumors showing signs of progression to MIBC were found predominantly in classes II and III and less often in class I. Sixteen MIBC tumors were analyzed together with NMIBC, and fourteen were categorized into class II, which indicates that there are similarities between MIBC and high-risk NMIBC class II [7]. NMIBC tumors with good prognosis, at an early stage and tumors exhibiting elevated expression of early cell cycle genes are categorized into class I. In contrast, NMIBC tumors at high stages and grades of disease with poor prognosis and tumors showing higher expression of late cell cycle genes belong to class II. Previous studies associated UC aggressiveness with overexpression of late cell cycle genes [11], a finding that is in agreement with the almost exclusive categorization of aggressive tumors into class II. Furthermore, class II tumors are associated with mutations in the *ERBB*, *MAPK/ERK*, and DNA damage response gene families. An APOBEC mutation signature is typical of class II tumors and is most likely caused by defects in DNA repair mechanisms [7].

Regarding the expression profiles of the three classes of NMIBC, both class I and II exhibit a balanced proportion of induced and repressed genes, whereas class III tumors show mainly repressed genes. In contrast to class I and II tumors, which show increased expression of uroplakins that are predominantly found in luminal or umbrella cells [7], class III tumors demonstrate a higher expression of *KRT5* and *KRT15*, which both mark undifferentiated (basal) cells [35]. Moreover, class II tumors show elevated expression of *KRT20*, which is highly linked to differentiated umbrella cells or CIS lesions [36], and *ALDH1A1, ADH1A2, PROM1* (*CD133*), *NES*, and *THY1* (*CD90*), which are cancer stem cell markers. Both class II and III tumors show enrichment in *KRT14*, and class III tumors also show enrichment in the expression of *CD44*, which are markers of basal cells and stem cells (Table 3) [7].

**Table 3:** NMIBC subtypes and their molecular features. Most of Hurst´s subtypes of Ta tumors are classified as class I, T1E1 subtype cases are classified as class I, IIb or III

The Aarhus group classification	Class I	Class IIa	Class IIb	Class III
Gen e expression	Uroplakins	Uroplakins *KRT20* *KRT14*	Uroplakins *KRT20* *KRT14*	*KRT5* *KRT15* *KRT14* *CD44* *GATA3*
Regulon activity		*FOXM1* *ESR2* *ERBB2* *ERBB3*	*ESR1* *FGFR1* *RARB* *STAT3* *PGR*	*AR* *GATA3*
Molecular feature	Early stage, high expression of early cell cycle genes	High expression of late cell cycle genes	The highest immune infiltration of NMIBC	Dormant state of disease
Prognosis	Good	The poorest	Poor	

Lund taxonomy applied to NMIBC	UroA	UroB	UroC	GU
Expression	Urothelial differentiation signature, *FGFR3*	*KRT5* *CDH3* *MYC*	*MYCL* *MYCN*	*MYCL* *MYCN*
Genomic alternation	*FGFR3* *KDM6A* *RAS* *TERT* *PIK3CA*	*RAS* *CDKN2A* deletion Loss of *TP53* *TERT* *PIK3CA*	*TP53* *TERT* *PIK3CA*	*RB1* *TP53* Loss of *TP53* Loss of* RB1* *E2F3* amplification *TERT* *PIK3CA*

T1E4 and part of T1E2 cases are classified as IIa. T1E3 cases are classified as class IIb

It can be said that in tumors of classes I and II, differentiation markers are expressed more often than in class III tumors; in addition, EMT transcriptional factors are expressed primarily by class II tumors. Ultimately, class I and II tumors can be considered luminal, while class III tumors are basal. Nevertheless, due to their similar luminal features, class I and II tumors exhibit differences in aggressiveness and should be treated accordingly [7].

Although class III tumors display gene expression signatures similar to basal-like MIBC (high *KRT5, KRT14, CD44* expression, and low *KRT20, PPARγ* expression) and Ta and T1 basal-like NMIBC tumors have been classified as class III, which may evoke the Ta disease progression pathway, class III tumors should not be considered precursors to the basal subtype of MIBC. The main reason for this distinction is that *GATA3* expression is upregulated rather than downregulated in class III tumors, which represents the major difference between basal MIBC and class III NMIBC. Tumors of class II, on the other hand, may evoke the CIS progression pathway, as these are typically high-risk luminal-like tumors. Class III tumors could be regarded as dormant because they exhibit low cell cycle activity and a low level of metabolism, demonstrate notable histone and chromatin remodeling activity, and overexpress various lnc-RNAs (e.g., *MIR31HG*, *NEAT1*, and *MALAT1*) [7]. As lnc-RNAs mediate gene silencing [37], their overexpression in class III tumors further supports the tumors being considered to represent a dormant state of disease [7].

Hedegaard’s classification was further supported and extended by the same research group, as they identified not three but four classes within NMIBC by subdividing class II into two subgroups: class IIa and class IIb. This research classified patients based on their likelihood of experiencing a recurrence-free survival (FRS) and progression-free survival (PFS), the ones with the highest RFS are assigned to class I, whereas those with the lowest PFS are categorized as class IIa, proceeded by class IIb. Class IIa tumors are linked with the expression of late cell cycle genes, genes linked with cell differentiation and DNA replication, and uroplakins. In contrast, class IIb tumors are linked with the expression of genes related to EMT and cancer stem cell marker expression, but class IIb tumors are not highly associated with cell proliferation gene expression. Class IIb tumors exhibit the highest rates of infiltration by immune cells compared with the remaining NMIBC classes [8]. Class IIb tumors might be sensitive to immunotherapy [38, 39], as class IIb tumors exhibit the highest levels of immune-checkpoint marker expression [8].

Elevated activity of *AR* and *GATA3* regulons is seen in class III tumors, whereas elevated *ERBB3, ESR2*, and *FOXM1* regulon activity can be seen in class IIa tumors. Class IIb tumors show the high activity of *PGR, RARB, STAT3, FGFR3*, and *ESR1* regulons (Table 3). With regard to epigenetic processes, research has revealed that class I tumors have more methylated gene promoters than those of class III. The highest numbers of single nucleotide variants are seen in class IIa tumors [8]. Immunochemical staining of GATA3 and CK5/6 as markers of luminal and basal differentiation, revealed that all of the analyzed NMIBC tumors were GATA3 positive, and almost one-third were CK5/6 positive. Furthermore, CK5/6-positive tumors were also GATA3-positive. In light of these findings, we should not consider NMIBC as luminal or basal by definition [40]. The strongest enrichment of CK5/6 expression can be seen in class III tumors, which also display high keratin 5 expression [8].

In general, class II can be considered to include high-risk tumors exhibiting numerous progression events. Tumors from class IIa are characterized by significant APOBEC mutation signatures and by a higher RNA-derived mutational load. In comparison, tumors from class IIb exhibit enrichment in stem cells and EMT marker expression as well as higher infiltration by immune cells, and they exhibit a lower risk of progression overall [8]. Hurst et al. [41] reached for another approach while analyzing and subtyping NMIBC tumors. In this study, they analyzed only Ta and T1 tumors first combined together and then also separately. Combined analysis of Ta and T1 tumors revealed the existence of 4 subtypes (E1-E4), which aligned well with the previously mentioned classification, with class I and class III being defined as E1 and E2 subtypes, class IIa as E3 and class IIb as E4. When analyzing only Ta tumors, based on copy number data and mutational features, they identified two genomic subtypes (GS1 and GS2) based mainly on one characteristic—loss of 9q, which prevailed in GS2. In GS2, there was a higher prevalence of *TSC1* mutations, which is in line with its location on chromosome 9q. On the other hand, GS1 showed a higher occurrence of *KMT2D* mutations, while GS2 exhibited a higher frequency of *KMT2A* mutations. In 70% of cases, either *FGFR3* mutations or *HRAS* mutations were present, but not both simultaneously. When considering transcriptional subtypes, within Ta tumors there were identified 3 expression subtypes, named TaE1-TaE3. The majority of GS1 tumors were found in TaE1 and TaE3, whereas TaE2 predominantly contained GS2 tumors and high-grade tumors. The best RFS was detected in TaE3 subtype. TaE3 exhibited the highest level of interferon signaling and there was a significant infiltration by various immune cell types. Moreover, TaE3 tumors exhibited heightened cytolytic activity. These findings strongly imply that the extended RFS observed in TaE3 subtype can be attributed to an intensified immune response against tumors.

The expression profile of the TaE1 subtype exhibited a heightened presence of genes implicated in the transcription of RNA and the synthesis of proteins. Additionally, it showcased an abundance of small nucleolar RNAs and the expression of transcriptional regulators associated with the differentiation of urothelial cells. On the other hand, the TaE2 subtype demonstrated a notable enrichment in the expression of genes related to late cell cycle processes, the response to hypoxia, glycolysis, the maintenance of cholesterol balance, and the metabolism of fatty acids. The TaE2 subtype displayed distinct characteristics compared to the TaT1 and TaT3 subtypes in terms of regulon activity. Specifically, it exhibited notable differences in the activity of regulons such as *E2F1*, *E2F2*, and *FOXM1.* Conversely, the TaT1 and TaT3 subtypes demonstrated higher activity in regulons associated with factors involved in urothelial differentiation, as well as the *AR*, *TP53*, and *TP63* regulons [41].

Within T1 tumors there were identified 4 subtypes based on mutational features, designed as T1CN1–T1CN4. T1CN1 demonstrated minimal copy number alterations, with only a few instances of chromosome 9 deletions, but there were more single nucleotide variants compared to other subtypes. On the other hand, T1CN3 predominantly exhibited losses rather than gains. In T1CN1 and T1CN2, *FGFR3* mutations were found to be more prevalent, while *TP53* mutations were more common in T1CN3 and T1CN4. Additionally, T1CN1 exhibited a higher frequency of *ERCC2* mutations compared to other subtypes. Mutations in genes associated with the DNA damage response were frequently detected in the T1E3 and T1E4 subtypes.

Same as within Ta tumors, also within T1 tumors were identified 4 different expression subtypes (T1E1–T1E4). T1E1 demonstrated the most favorable PFS, while E1T4 the poorest PFS among subtypes. *RB1* mutation and *E2F3* amplification were exclusively detected in T1E3 and T1E4. The occurrence of *TP53* mutation was elevated in T1E2, T1E3, and T1E4, which exhibited a higher frequency of copy number alterations. The T1E1 and T1E2 subtypes exhibited a notable increase in the expression of genes involved in the initiation of translation, protein targeting, and the biogenesis of ribosomes. In contrast, the T1E3 and T1E4 subtypes displayed enrichment in the expression of genes associated with the immune system and inflammatory response. Moreover, both the T1E2 and T1E4 subtypes showed a shared enrichment in genes related to DNA repair, replication, and various metabolic processes. The T1E2 and T1E4 subtypes also demonstrated an upregulation of the *E2F1* and *FOXM1* regulons, while the activity of *PPARγ* was reduced in the T1E3 and T1E4 subtypes. Many of Ta tumors were classified as class I from the previous classification system. T1E1 cases belonged to class I, IIb or III, while T1E4 and part of T1E2 fell into class IIa. T1E3 subtype was associated with class IIb [41].

Study by Marzouka et al. [42] applied Lund classification [12, 13], which was originally created for both, NMIBC and MIBC tumors, on cases from the two previously mentioned classification systems—the Aarhus group classification [7, 8] and Hurst classification [41]. The Lund classification sets itself apart from other classification systems by focusing solely on categorizing tumors based on the characteristics of cancer cells, excluding non-tumor cells from the analysis. This approach makes Lund taxonomy unique, as any other of the mentioned classifications for NMIBC excluded non-tumor cells from their analyses [12, 13]. When Lund taxonomy was applied to the Aarhus group cases, 507 out of 535 tumors were classified as urothelial, 23 tumors were categorized as GU. Furthermore, within the cohort, three samples were classified as Ba/Sq, and two samples were categorized as mesenchymal-like. Urothelial tumors were further divided into subgroups: 443 as UroA, 41 as UroB, and 23 as UroC. Out of the 217 cases from Hurst study, classification was conducted using the Lund system, resulting in the identification of 183 UroA cases, 6 UroB cases, 15 UroC cases, 12 GU cases, and one case classified as the Ba/Sq subtype. UroA class was defined by the highest levels of expression of urothelial differentiation signature, expression of *FGFR3*, and also by frequent mutations in *FGFR3. KDM6A* was mutated predominantly in UroA subtype and *PPARγ* mutations were observed solely in UroA tumors, and they occurred at relatively low frequencies. *TERT* and *PIK3CA* genes were mutated in all classes, while *RAS* mutations were found only in UroA and UroB. *STAG2* mutations were completely absent in UroC subtype. UroB class showed the lowest expression of urothelial differentiation signature, almost no detected presence of *UPK3A* or *KRT20* expression, but high expression of *KRT5* and *CDH3*. UroB tumors showed expression of the transcription factor *MYC*, while *MYCL* and *MYCN* were not detected. Conversely, UroC and GU tumors displayed the opposite pattern, with expression of *MYCL* and *MYCN*, but not *MYC*. UroC and GU tumors did not exhibit any homozygous deletions of *CDKN2A*, whereas UroB tumors displayed frequent occurrences of such deletions. Expression of *FGFR3* was nearly absent in UroC and GU subtypes, and both classes showed low expression of *KRT5* and *CDH3*. Infiltration by immune and stromal cells was found predominantly within UroB and UroC classes. Tumor progression was linked with GU class, followed by UroB, and tumors of high grade were classified as GU or UroC. *RB1* mutations were nearly non-existent in all tumor subtypes, except for GU. Conversely, *TP53* mutations were primarily observed in UroC and in GU tumors. For GU tumors there were typical *TP53* losses (shared with UroB), RB1 losses, and *E2F3* amplifications (Table 3). The analysis uncovered significant genomic alterations in the UroC and GU subtypes, notably, nearly all UroC and GU tumors were found to be triploid [42]. Molecular features of Aarhus cases and Hurst cases corresponded well. The UroC subtype stood out as the only notable divergence, with the Hurst UroC cases displaying higher rates of *TP53* and *CDKN2A* deletions, as well as *CCND1* amplifications, in comparison to the Aarhus UroC cases [42]. These findings confirm that Lund’s classification is useful and applicable not only for MIBC, but also for NMIBC [11–13, 42].

## Discussion

The first attempts to classify cancer based on molecular signatures were made in breast cancer, where it was first described that tumors display a typical luminal or basal phenotype characterized by molecular changes [43]. A deeper molecular analysis of UC revealed there are several distinct subtypes, each with characteristic properties. That gave rise to a novel molecular classification of UC. Currently, the standard for classifying UC is the TNM classification system [4]. The treatment primarily relies on clinical parameters (TNM staging), since there are no precise treatments available that target specific vulnerabilities of the tumor. [44].

Recent evidence suggests that molecular classification of UC may provide more precise stratification that could impact treatment and patient survival. Molecular differences between UC subtypes underlie biological differences and tumor behavior, which consequently lead to differences in aggressiveness, prognosis, and progression. Evidence is clear that NMIBC and MIBC are heterogenous groups of tumors and that the TNM classification system is not sufficient for the characterization of this heterogeneity. Both NMIBC and MIBC tumors exhibit varied responses to therapy; thus, utilization of detailed molecular classification information could enhance clinical understanding and aid in the management of UC patients. Because of the differences in the biological properties of UC subtypes, it is assumed that subtypes will exhibit differences in sensitivity or resistance to therapy. Molecular classification of UC tumors has the potential to significantly impact and influence the clinical management of MIBC, enabling the tailoring of more precise treatments and assessment of treatment response, especially with regard to neoadjuvant chemotherapy (NAC) of MIBC. The administration of NAC leads to better patient survival rates and improvement in pathological downstaging; however, a major response, characterized by the lack of muscle-invasive disease and/or metastasis to lymph nodes, is observed in only about 40% of patients [45]. Non-responders to NAC usually do not obtain a clinical benefit; quite contrary, they experience severe toxicity and may have a postponement in definitive local therapy [46]. Thus, the potential impact of molecular subtyping may be in guiding more precise treatment and selection of optimal therapy. For instance, the initial proposal of the idea that distinct molecular subtypes could be indicative of the response to NAC was introduced by Choi et al. [9]. In this study, patients who were diagnosed with “p53-like” tumors exhibited a reduced rate of response to cisplatin-based NAC combination. Nevertheless, further validation of this observation in a large patient population has not been conducted. By analyzing a large group of patients, the study by Seiler et al. presented additional evidence demonstrating that the outcome after NAC differs depending on the molecular subtype [47]. These findings provide evidence in favor of the practical application of identifying molecular subtypes of MIBC, with the authors concluding that individuals with basal tumors experience the greatest advantage from NAC. In contrast, regardless of the treatment approach, patients with nonimmune-infiltrated luminal tumors had the most favorable outcome in terms of their prognosis, suggesting that NAC is not beneficial for these patients. The limitation of that study was its retrospective design, but it clearly showed an association between molecular subtype and NAC treatment response [47]. Hence, a comprehensive multi-omics analysis of 300 MIBC patients treated with chemotherapy was performed to identify molecular alterations linked to the response to treatment [48]. In this study, authors used integration of genomic and transcriptomic data, and stratified patients into clusters based on their varying probabilities of responding to cisplatin-based treatment (basal/squamous gene expression subtype is linked to a diminished response to chemotherapy, whereas immune cell infiltration and elevated PD-1 protein expression are indicative of favorable treatment response) [48].

In clinical trial settings, immune-checkpoint inhibitors are employed for patients with MIBC [49]. Mariathasan et al., were the first to identify major determinants of the clinical outcome of metastatic UC treatment with atezolizumab (an immune-checkpoint inhibitor) [38]. The first clinical data were published within the PURE-01 trial, which showed promise in identifying patients who are more likely to benefit from NAC, while also avoiding the possible harmful effects of treatment for others [50, 51].

A recent study of 601 patients revealed that the use of a molecular subtyping assay resulted in notable benefits from NAC for individuals with non-luminal tumors, while those with luminal tumors had only a slight improvement in survival [52]. In contrast, conflicting results have been reported which demonstrate that the response to atezolizumab or cisplatin-based chemotherapy does not show any correlation with the molecular subtype of UC [53], which could be due to the impact of transurethral resection of the bladder tumor before therapy and differences in immune scores [54].

Furthermore, other papers described links between tumor microenvironment and response to NAC [55, 56], immunotherapy [53, 54, 57], response to the combination of both therapies [58], and finally, a study examining intratumoral heterogeneity and response to treatment [59]. Indeed, a significant body of evidence indicates that treatment response depends not only on the molecular subtype of BC but also on tumor microenvironment and intratumoral heterogeneity.

Regarding NMIBC, the potential benefit and clinical utility of molecular subtyping may be in the more-accurate prognostication of recurrence and progression. In a cohort of 834 patients, Lindskrog et al. showed that disease aggressiveness is associated with immune cell infiltration, genomic modifications, and transcriptomic classes. Furthermore, the degree of genomic modifications in NMIBC serves as a prognostic indicator for the likelihood of progression and recurrence, irrespective of other influencing factors; therefore, tumors displaying significant chromosomal instability need to be treated as high-risk category tumors, irrespective of their histopathological results [8]. A recent study suggested the effectiveness of the prognostic significance of the molecular signature-based subtype predictor (MSP888) and the authors compared its effectiveness to the risk scores of the 2021 EAU, CUETO, and EORTC [60]. MSP888 is based on molecular signatures, which consists of three distinct subtypes, MSP888 was proven to distinguish NMIBC patients with varied prognoses and responses to bacillus Calmette-Guérin (BCG) treatment in their previous study [61]. The predictive ability of the MSP888 classifier in determining the prognosis of NMIBC was found to be very high, proposing that systems that classify tumors based on molecular features are superior in accuracy compared to risk scores that rely on clinicopathological traits. These results clearly indicate a shift in the paradigm based solely on the TNM classification system.

The majority of T1 BTs are managed using BCG therapy. In the event of recurrence or progression, radical cystectomy is employed, and postponing intervention is linked to decreased survival rates [3]. The mechanisms underlying BCG response have been investigated in addition to gene expression patterns and the tumor microenvironment, and these studies showed that patients who do not respond to BCG treatment exhibit elevated levels of *PD-L1* expression in their tumors compared to those who respond to BCG therapy [62], implying that the tumor microenvironment before treatment is a critical determinant of the BCG response mechanism [8]. A study by Robertson et al. investigated the molecular heterogeneity of primary T1 tumors by performing RNA sequencing on 73 samples, with a primary objective of evaluating recurrence following BCG treatment. Five different molecular subtypes of T1 tumors were discovered, which seemed to correlate with two primary categories of regulon activity and different responses to BCG treatment [63]. These studies indicate that molecular subtyping may shed light on the treatment and selection of BCG non-responders, leading to more-precise treatment regimens in the future.

Molecular subtyping and gene expression has enhanced our comprehension of UC biology in contrast to the conventional classification system, as it mirrors the inherent properties of tumors and anticipates the prognosis and responsiveness to treatment for patients suffering from UC. The critical role of precise identification of MIBC patients who are most likely to achieve optimal outcomes with neoadjuvant checkpoint inhibitor therapy, either as monotherapy or in conjunction with NAC, cannot be overstated in guiding future treatment approaches. Given the enormous amount of information in recent years, using “multi-omics” analysis, differences in intratumor environment, and heterogeneity, artificial intelligence may appear to be a “game changer” to model and tailor treatment in the near future [64, 65].The molecular classification of UC has not yet been officially approved by any oncology society and remains unstandardized, as several groups have created nomenclatures that may be confusing and unsuitable for clinical utilization. There is a need for consensus that takes into account molecular, biological, clinical, and prognostic features of tumors, with close cooperation between expert societies (e.g., EAU, AUA) based on well-designed clinical trials and evidence-based medicine. Nevertheless, molecular subtyping represents a path toward personalized and precision medicine that may change the guidelines of major medical societies in the near future.

## Conclusions

Classification based on gene expression and genomic alternations enhances understanding of tumor behavior and may aid in tailoring treatments, thus improving the prognosis of UC patients. Achievement of classification consensus could pave the way for well-designed prospective clinical trials that include molecular subtyping, which could change current guidelines and treatment approaches for UC patients.

## Data Availability

No additional data are available.

## Abbreviations

BCG: Bacillus calmette-guérin,
Ba/Sq: Basal/squamous,
Ba/Sq: Basal/squamous cell carcinoma-like
UC: Urothelial carcinoma
BT: Bladder tumor
CIS: Carcinoma in situ
EGFR: Epidermal growth factor receptor
EMT: Epithelial-to-mesenchymal transition
ESR2: Estrogen signaling receptor
GU: Genomically unstable
LumNS: Luminal non-specified
LumP: Luminal papillary
LumU: Luminal unstable
mes-like: Mesenchymal-like
MIBC: Muscle-invasive bladder cancer
NAC: Neoadjuvant chemotherapy
Ne-like: Neuroendocrine-like
NMIBC: Non-muscle-invasive bladder cancer
OS: Overall survival
PPARγ: Peroxisome proliferator activator receptor-gamma
PFS: Progression-free survival
RFS: Recurrence-free survival
Sc/Ne: Small cell/neuroendocrine-like
SCCL: Squamous cell carcinoma–like
TNM: Tumor node metastasis
UroA: Urobasal A
UroB: Urobasal B
UroC: Urobasal C
